# *Staphylococcus aureus* Protein A Induces Human Regulatory T Cells Through Interaction With Antigen-Presenting Cells

**DOI:** 10.3389/fimmu.2020.581713

**Published:** 2020-09-30

**Authors:** Julia Uebele, Katharina Habenicht, Olga Ticha, Isabelle Bekeredjian-Ding

**Affiliations:** Division of Microbiology, Paul-Ehrlich-Institut, Langen, Germany

**Keywords:** *Staphylococcus aureus*, Treg—regulatory T cell, Ig-binding proteins, protein A, tolerance, immune suppression, immune evasion, human

## Abstract

Despite continuous exposure and development of specific immunity, *Staphylococcus aureus* (Sa) remains one of the leading causes of severe infections worldwide. Although innate immune defense mechanisms are well understood, the role of the T cell response has not been fully elucidated. Here, we demonstrate that Sa and one of its major virulence factors protein A (SpA) induce human regulatory T cells (Tregs), key players in immune tolerance. In human PBMC and MoDC/T cell cocultures CD4^+^CD25^+^CD127^dim^ Tregs were induced upon stimulation with Sa and to a lower extent with SpA alone. Treg induction was strongly, but not exclusively, dependent on SpA, and independent of antigen presentation or T cell epitope recognition. Lastly, soluble factors in the supernatant of SpA-stimulated MoDC were sufficient to trigger Treg formation, while supernatants of MoDC/T cell cocultures containing Sa-triggered Tregs displayed T cell suppressive activity. In summary, our findings identify a new immunosuppressory function of SpA, which leads to release of soluble, Treg-inducing factors and might be relevant to establish colonization.

## Introduction

The human commensal *Staphylococcus aureus* (Sa) is a major pathogen and leading cause of nosocomial infections, resulting in tens of thousands of deaths worldwide and causing billions of dollar economical damage per year (1–3). About 30% of the human population are colonized (4). The increasing antibiotic resistance of Sa strains [so called MRSA, (5)], resulting in long-lasting infections, illustrates the urgent need for a protective vaccine. However, despite promising approaches during the last decades and successful *in vivo* studies, all vaccine attempts have failed to date (6–8). One of the many reasons is the lack of essential pieces in understanding the complex human immune response against this pathogen.

For many years, research focused on the humoral immune response against Sa since antibodies against this bacterium can be found in asymptomatically colonized individuals as well as in patients (9, 10). However recently, more and more research has been dedicated to T cell-mediated immunity, demonstrating that this arm of the immune system plays an important role in Sa clearance. Several models of infection in mice and in human have shown that CD4^+^ T cells are important for the immune response to Sa, as reviewed elsewhere in detail (6, 11, 12). Infections were more severe in *ifnγr*-deficient mice (13) and patients with Th1-deficiencies are more prone for infection (14). Lin *at al*. showed that protection in vaccinated mice was caused by secretion of IFNγ and IL-17 from CD4^+^ T cells (15). Several others also proved that Th17-mediated immunity is essential during Sa infection (16–19). Furthermore, we and others demonstrated that a human memory T cell repertoire exists in healthy individuals (20–22), underlining the importance of previous exposure.

One of the many reasons, Sa is such a potent immune activator is its vast variety of virulence factors, among them staphylococcal protein A (SpA). This cell-wall anchored protein (23) is expressed by almost all Sa isolates and highly abundant on the staphylococcal cell wall (24). It harbors 4-5 Ig-binding domains (25) and by binding the Fcγ domain of IgG (26) it can prevent opsonization (24) and FcR-mediated phagocytosis (27). Additionally, SpA binds the B cell receptor (28) of VH3^+^ B cells [30%–60% of B cells in human (29)]. This leads to B cell proliferation, apoptosis (30) and with the help of plasmacytoid dendritic cells, to formation of regulatory B cells (Bregs). These cells secrete IL-10, a cytokine associated with suppression of antigen presentation and T cell responses (31, 32).

Another important cell subset to prevent and control overshooting immune responses are regulatory T cells [Tregs, (33)]. Through secretion of immunosuppressive cytokines such as IL-10 and TGFβ, consumption of immunostimulatory cytokines, secretion of cytolytic factors and interaction with antigen-presenting cells (APC) they maintain immune homeostasis (34). Due to their strong immunosuppressive properties they are being evaluated for treatment of autoimmune diseases (35). CD4^+^ Tregs are described as Forkhead-Box-Protein P3^+^ (FoxP3), CD25^+^ [IL-2 receptor α chain), and CD127^dim/-^ (IL-7 receptor, (36)].

Several groups demonstrated a potential role of Tregs in post-infectious arthritis following Sa infection (37–39) and in atopic dermatitis (40). Furthermore, the Sa virulence factor phenol-soluble modulin (PSMα) was shown to modulate human and mouse APC surface marker expression and cytokine secretion to induce Treg *in vitro* and *in vivo* (41–43). Moreover, it has been shown that staphylococcal superantigens induce human Tregs in PBMC (44) and convert peripheral CD4^+^CD25^-^ T cells to a regulatory phenotype with suppressive function (45, 46).

In earlier studies, we saw that SpA induces Treg-associated cytokines *in vitro* (20). This study aimed to investigate the potential of this B cell superantigen in the induction of human Tregs.

## Materials and Methods

### Bacteria

*Staphylococcus aureus* WT strain SA113, *spa*-deficient mutant SA113 Δ*spa* and SA113Δ*lgt* lacking TLR2 activity (provided by Friedrich Götz, Tübingen) were grown on Columbia blood agar plates (supplemented with 20 µg/ml Erythromycin for mutant strains) overnight at 37°C.

### Reagents, Stimuli, and Antibodies

Stimulation of cell culture was carried out with 1 μg/ml protein A (SpA, isolated from *S. aureus* SAC, GE Healthcare, Uppsala, Sweden), 1 µg/ml recombinant SpA (Sigma-Aldrich, Munich, Germany), 100 ng/ml synthetic lipopeptide P3C (Pam3CSK4, EMC, Tübingen, Germany) or anti-CD3/CD28 microbeads (Miltenyi Biotech, Bergisch-Gladbach, Germany).

Microbeads used for cell isolation *via* AutoMACS and recombinant cytokines (IL-4 and GM-CSF) were obtained from Miltenyi Biotech (Bergisch-Gladbach, Germany).

Antibodies used for flow cytometry, purity determination and cell sorting were purchased from BD Biosciences, Heidelberg, Germany, if not indicated otherwise: CD4 PerCP, CD4 PE, CD3 BV605 (BioLegend, U.S.), CD3 AF700 (BioLegend, U.S.), CD25 APC (BioLegend, U.S.), CD25 FITC, CD127 BV421, CD127 AF647, FoxP3 BV421 (BioLegend, U.S.), CCR4 PE-Cy7 (BioLegend, U.S.), ICOS BV605 (BioLegend, U.S.), CTLA4 BV421 (BioLegend, U.S.), PD-1 PE (BioLegend, U.S.) and CD14 V450. Viability staining was performed with the LIVE/DEAD Fixable dead Cell stain Kit (Thermo Fisher Scientific, U.K.).

### Isolation of PBMC and T Cells

Use of human peripheral blood mononuclear cells (PBMC) from buffy coats was approved by the local institutional review board (Ethics committee of the Medical Faculty of the University of Frankfurt, Germany, #154/15). Buffy coats of anonymized healthy donors were obtained from the German Red Cross South transfusion center (Frankfurt am Main, Germany).

PBMC were isolated by Pancoll gradient centrifugation (PAN-Biotech, Aidenbach, Germany). T cells were isolated by positive selection with anti-CD4 microbeads *via* AutoMACS. Purity was determined by CD4 antibody and was always ≥97% (**Figure S1**).

### Generation of MoDC and Cocultures With Autologous T Cells or PBMC

MoDC were generated by positive CD14^+^ isolation *via* AutoMACS and culture in medium containing 50 ng/ml human GM-CSF and 20 ng/ml human lL-4. Fresh medium supplemented with 100 ng/ml GM-CSF and 40 ng/ml IL-4 was added on day 3 and MoDC were cultured for 6d, as described earlier (20). Remaining PBMC were frozen in 50% FCS and 50% freezing medium (RPMI 1640, supplemented with 20% FCS and 20% DMSO (both from Sigma-Aldrich, Munich, Germany)) for subsequent isolation of autologous T cells.

If not stated otherwise, culture of PBMC, MoDC/PBMC cocultures or MoDC/T cell cocultures was carried out under Ig-free conditions in RPMI 1640 (Gibco by Life science, Darmstadt, Germany), supplemented with 5% Xerumfree (TNC Bio, Eindhoven, Netherlands) as serum-free alternative, 1% penicillin/streptomycin (10,000 IU/ml and 10,000 μg/ml) and 1% 200 mM L-Glutamine (all from Biochrom AG, Berlin, Germany). For some experiments, instead of Xerumfree, 5% heat-inactivated human serum (Biochrom AG, Berlin, Germany) or chicken serum (in house) was added. In this case, SpA was incubated with the indicated media for 2 h rolling before being added to the cells, to ensure blocking of SpA’s Ig-binding sites.

1*10^5^ PBMC or 1*10^5^ CD4^+^ T cells were seeded in 96 well plates alone or in coculture with 2*10^4^ MoDC in 200 µl medium total. Cells were stimulated with bacteria at a ratio 1:10 (MOI=10, determined by McFarland 1) or 1 μg/ml SpA and incubated for 5d at 37°C and 5% CO_2_ for Treg induction or cytokine measurement.

For generation of ivt *spa* mRNA and MoDC transfection, see (20) for details.

For determination of MoDC-derived cytokines or generation of MoDC SN for T cell stimulation, MoDC only were seeded with a density of 1*10^5^/96 well in 200 µl medium total and stimulated with the indicated stimuli for 24h at 37°C and 5% CO_2_. The plate was centrifuged, SN taken and used for cytokine determination by MagPix XMAP technology.

For analysis of Treg-inducing capacity, SN was re-centrifuged for 6 min at 1300 rpm and frozen for minimum 1 h at -20°C before being added to T cells, to avoid any contamination by remaining MoDC.

### Flow Cytometry and Cell Sorting

Flow cytometry was performed at a FACS LSRII SORP (BD Biosciences, Heidelberg, Germany). Data was analyzed by Kaluza 2.1 Software (Beckman Coulter, U.S.).

For cell sorting, PBMC were isolated from buffy coats and stored overnight at 4°C in PBS, supplemented with 5% Xerum free. The next day, cells were washed, stained with CD4 PerCP, CD3 BV605, CD25 FITC, CD127 AF 647 and Tregs were depleted with a 70 μm nozzle with BD FACSAria Fusion (BD Biosciences, Heidelberg, Germany), using the BD FACS Diva software version 8.0.1. After sorting, cells were collected in tubes coated with 10% BSA overnight (to increase cell recovery rate) and containing RPMI with 5% Xerum free, 1% Pen/Strep, and 1% L-Glutamine. As control, full PBMC were also stained and sorted, but Tregs were not depleted (for sorting scheme see **Figure S7A**). Purity of sorted cells was confirmed by re-analysis and was >90% (**Figure S7B**).

After sorting, cells were stimulated with the indicated stimuli, incubated for 5d and re-stained with CD25 FITC, CD4 PerCP, and FoxP3 BV421, using the FoxP3 Fix/Perm Buffer Set (BioLegend, U.S.) and following the manufacturer’s protocol.

### Suppression Assays

For generation of Treg-enriched SN, 6*10^5^ CD4^+^ T cells were seeded in 24 well plates together with 1.2*10^5^ MoDC in 250 µl medium each (RPMI with 5% human serum, 1% Pen/Strep, 1% L-Glutamine) and were stimulated with Sa WT MOI= 10. Culture in 24 well plates was carried out in a total volume of 1 ml.

To avoid remaining viable bacteria in the SN during subsequent stimulation, Sa was heat-inactivated by incubation at 60°C for 30 min (HISA). Heat-inactivation was confirmed by spreading bacterial suspension on Columbia blood agar plates and incubation at 37°C overnight. No bacterial growth was detected.

After 5d incubation, plates were centrifuged, induction of Tregs was confirmed by flow cytometry as described above and SN was collected, re-centrifuged and frozen once to lyse remaining cells.

Next, to determine suppressive capacity of Treg-enriched SN, autologous CD4^+^ T cells were isolated from frozen PBMC by AutoMACS as described above, stained with CFSE (Thermo Fisher Scientific, U.K.) and seeded in 100 µl medium (10% human serum, 2% Pen/Strep, 2% L-Glutamine) at a density of 5*10^5^/ml. For cell stimulation, 0.5 µl anti-CD3/CD28 beads (≙100.000 beads; cell: bead ratio 1:1) and either 100 µl Treg-enriched SN (considered serum-free) or 100 µl RPMI pure were added to the autologous T cells. Cell proliferation was assessed after 4d by measurement of viable, CFSE^low^ cells by flow cytometry.

### Cytokine Measurements

Cytokines in supernatants from MoDC cultures or MoDC/T cell cocultures were quantified by the ELISA Multiplex MagPix XMAP technology (Luminex, 640 Austin, U.S.), detecting and quantifying multiple cytokines in one assay. The Milliplex Human Th17 Magnetic Bead Panel Kit (Merck Millipore, Darmstadt, Germany) was used to analyze cytokine levels in supernatants of MoDC stimulated for one day with SpA or ivt mRNA. T cell cytokines in MoDC/T cell cocultures stimulated with bacteria for 5d were quantified using the Bio-Plex Pro Human Cytokine 17-plex Assay (Bio-Rad Laboratories GmbH, Munich, Germany).

### Statistical Analysis

Statistical analysis of results was carried out using GraphPad Prism 8.01 (Graphpad Software Inc. San Diego, USA). If not stated otherwise, following testing for normal distribution (Shapiro-Wilk-Test), paired two-tailed Student’s t-test was used, treating samples as paired data points. One-way ANOVA with LSD was used for testing multiple groups. Each experiment was carried out at least in duplicates in at least two independent experiments. Results were considered statistically significant at *p < 0.05, **p < 0.01, ***p < 0.001, and ****p < 0.0001. p ≥ 0.05 = not significant (ns).

## Results

### *S. aureus* Induces T Cell-Associated Cytokines in Cocultures With MoDC in a SpA-Dependent Manner

The immunostimulatory effect of SpA on B cells is well known. However, so far, there is only few data on this important virulence factor on the activation of T cells. Previously, we described the induction of IL-2, G-CSF, and IL-10 in cocultures of monocyte-derived dendritic cells (MoDC) and T cells upon SpA stimulation (20). These cytokines are associated with immunosuppression and/or Treg development.

**Figure 1:**
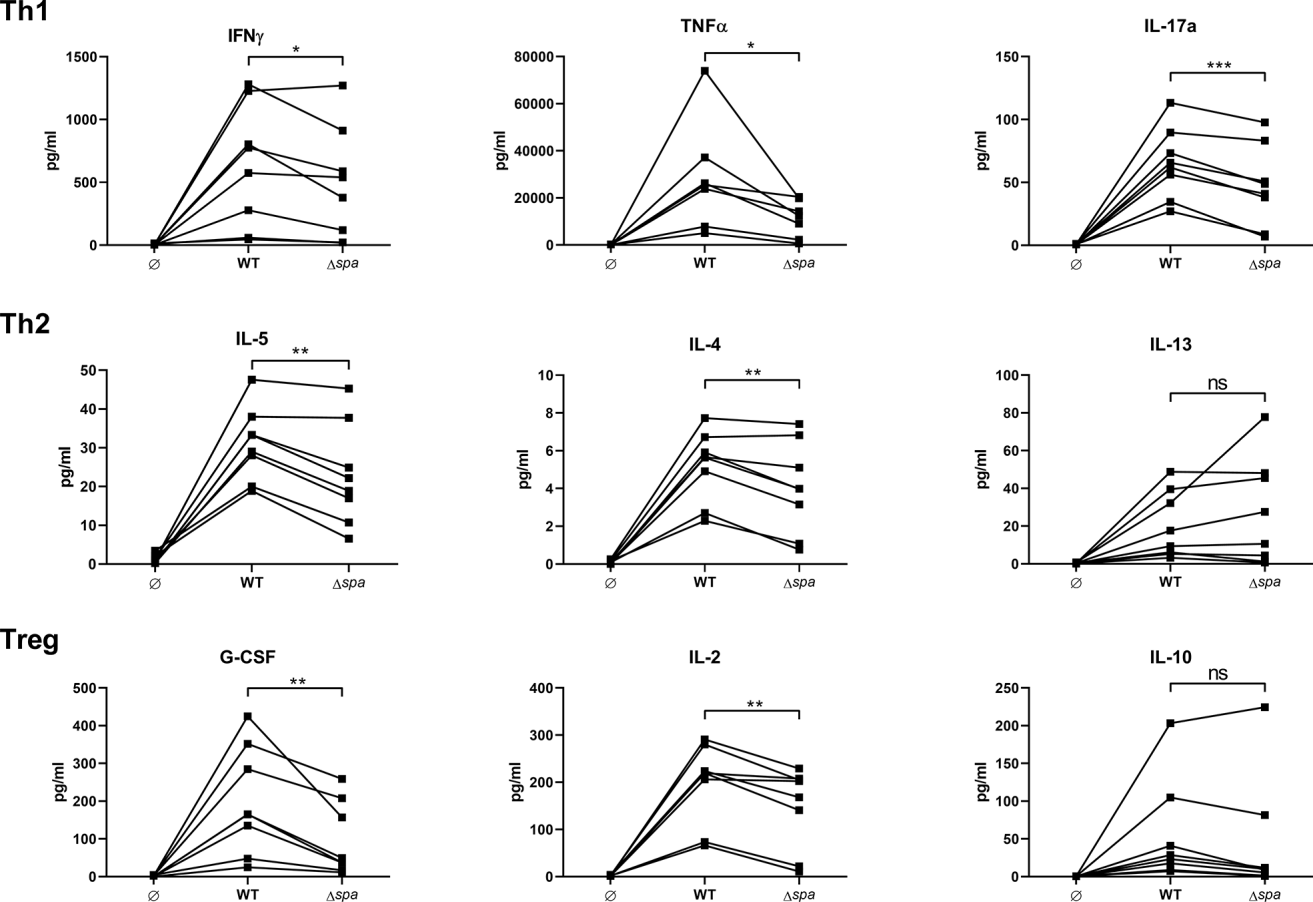
Staphylococcal protein A (SpA) induces T cell cytokine secretion. MoDC/CD4^+^ T cell cocultures were stimulated with SA113 WT or *spa*-deficient strain (Δ*spa*). Th1 (upper row), Th2 (middle row), and Treg (lower row) associated cytokines were analyzed in 5d supernatant by Magpix, a multiplex ELISA technology. N = 8 different donors of four independent experiments are displayed as individual, linked points. * and ** indicate statistical significance: *p < 0.05, **p < 0.01.

To analyze the impact of SpA on this cytokine profile, we stimulated human MoDC/CD4^+^ T cell cocultures with viable Sa (SA113 WT) or a *spa*-deficient mutant (SA113 Δ*spa*). After incubation for 5d, cytokine secretion was analyzed in the supernatants by MagPix, a multiplex ELISA system (**Figure 1**).

Overall, the wild type strain led to higher secretion of almost all detected cytokines. There was a significant decrease in Th1/Th17 cytokines (IFNγ, TNF and IL-17) when cells were stimulated with the SA strain lacking SpA expression (*Δspa*). Albeit the Th2 response was generally less pronounced, Δ*spa* induced significantly less IL-5 and IL-4. The Treg-associated and immunosuppressive cytokines G-CSF and IL-2 were also significantly decreased after *Δspa* stimulation. IL-10 levels were low, and even less in the absence of SpA expression. The same tendency was seen with IL-12p40, IL-1β and IL-6 (**Figure S2**). Thus, it was demonstrated that SpA is responsible for the strong immunostimulatory reactivity to Sa in MoDC/T cell cocultures.

### *S. aureus* and SpA Induce Human Tregs

**Figure 2:**
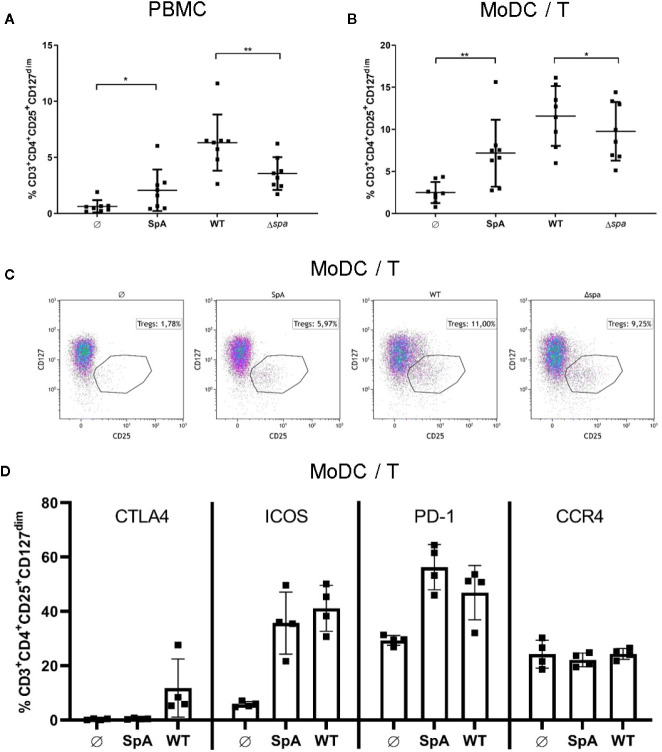
Staphylococcal protein A (SpA) is crucial for Treg induction. **(A)** peripheral blood mononuclear cell (PBMC) or **(B)** monocyte-derived dendritic cell (MoDC)/T cell cocultures were stimulated with SpA, SA113 WT or Δ*spa* bacteria. After 5d cells, were stained for viability and CD3^+^CD4^+^CD25^+^CD127^dim^ and were analyzed by flow cytometry. Results are shown from four independent experiments as single values of n=8 different donors with ± SD. Statististical significance is provided by * (*p < 0.05) and ** (**p < 0.01). **(C)** Induction of CD3^+^CD4^+^CD25^+^CD127^dim^ Tregs in MoDC/T cell cocultures upon stimulation with SpA, SA113 WT or Δ*spa*. Dot plots of one representative donor are shown. **(D)** CD3^+^CD4^+^CD25^+^CD127^dim^ Tregs in MoDC/T cell cocultures upon stimulation with SpA or SA113 WT were analyzed for CTLA4, ICOS, PD-1 and CCR4 expression after 5d of culture by flow cytometry. Results are shown from two independent experiments as single values of n=4 different donors with ± SD.

Next, we wanted to analyze whether SpA induces not only Treg-associated cytokines, but also Tregs. We therefore stimulated PBMC or MoDC/T cell cocultures with SA113 WT, SA113 Δ*spa* or SpA protein only. Experiments were carried out in media lacking immunoglobulins to prevent blocking of the SpA Ig-binding sites. On day 5, we stained cells for CD3^+^CD4^+^CD25^+^CD127^dim^ expression (**Figure S3**) and analyzed them by flow cytometry. Indeed, SpA induced a significant proportion of Tregs compared to the unstimulated control in PBMC (**Figure 2A**) and MoDC/T cell cocultures (**Figures 2B, C**). Interestingly, stimulation of PBMC with full bacteria induced an even higher percentage of Tregs, which was significantly reduced when bacteria lacked *spa* expression. Nevertheless, this proportion was still higher than induction of Tregs by SpA alone, indicating that additional immunostimulatory signals are important for induction of this cell type. This induction pattern was reproducible in MoDC/T cell cocultures (**Figure 2B**). Furthermore, we did not see differences in Treg levels when PBMC were stimulated with SA113 Δ*lgt*, lacking TLR2-activating lipoproteins, compared to SA113 WT (**Figure S4**).

To analyze the SpA and SA-induced Tregs in more detail, we stained the Treg with anti-CTLA4, anti-PD-1, anti-ICOS, and anti-CCR4 antibodies and quantified expression of these markers by flow cytometry (**Figure 2D**). The results showed that ICOS and PD-1 expression is induced upon formation of Tregs after stimulation with SpA protein or SA113 WT bacteria. Upregulation of CTLA4 was lower and only observed with bacterial stimulation. By contrast, CCR4 expression was not altered by SpA or SA113 WT stimulation. We conclude that SpA-dependent Treg induction promotes expression of ICOS and PD-1 but has little effect on CTLA4 and CCR4 expression.

### The Induction of Tregs by SpA Is Independent on Antigen Presentation

Next, we asked whether SpA has to be presented by APC or whether it directly acts on T cells. To this end, we choose a technical approach where antigen is delivered as mRNA to the dendritic cells. The *spa* gene was *in vitro* transcribed (ivt) and APCs were transfected with ivt mRNA. Upon uptake by MoDC, ivt mRNA is translated, antigen processed and presented to T cells. As control of unspecific stimulation by ivt mRNA nonantigen encoding mRNA (NC mRNA), as well as only the transfection reagent lipofectamine (LF) were used. In comparison to native SpA, Treg induction by ivt mRNA in MoDC/T cell cocultures was assessed after 5d by flow cytometry.

**Figure 3:**
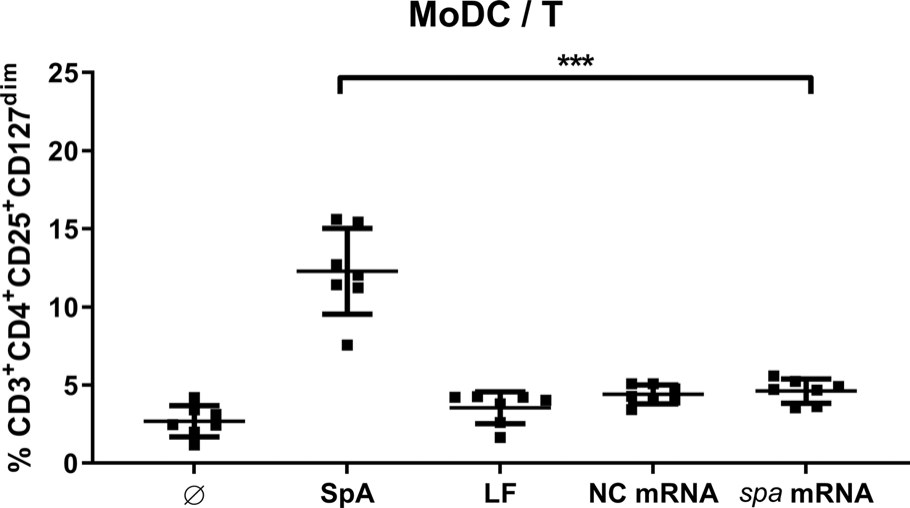
Native protein conformation of SpA is mandatory for Treg induction. Monocyte-derived dendritic cell (MoDC) were stimulated with staphylococcal protein A (SpA) or transfected with 100 ng mRNA (encoding *spa* or noncoding, NC) in cocultures with CD4^+^ T cells. As transfection control served lipofectamine (LF). After 5d, cells were stained for viability, CD3^+^CD4^+^CD25^+^CD127^dim^ expression and were analyzed by flow cytometry. Results are shown from three independent experiments as single values of n = 7 different donors with ± SD ***p< 0.001.

As displayed in **Figure 3**, Tregs were only induced when antigen was delivered as protein, whereas Treg induction in conditions with ivt mRNA and LF remained at unstimulated levels. Results could be reproduced in cocultures of MoDC and PBMC (**Figure S5**). Please note, that although ivt mRNA did not induce Tregs, cytokine secretion could be measured in MoDC cultures upon transfection, confirming that cell stimulation itself by transfection was successful (**Figure S6**). This led to the conclusion, that the native protein conformation of SpA is necessary for Treg induction and that processing and presentation of the antigen is not sufficient.

### SpA-Induced Treg Formation Is Blocked in the Presence of Human Immunoglobulins

Since native SpA’s most prominent characteristic is its Ig-binding capacity, we addressed this issue by adding different sera in the culture media to block the Ig-binding sites. We analyzed culture in medium supplemented with chicken serum or human serum. SpA does not bind chicken Ig (47), but human Ig with high affinity. All sera were heat-inactivated prior use and SpA was incubated 2 h with the media containing 5% of the indicated serum before being added to the cells.

**Figure 4:**
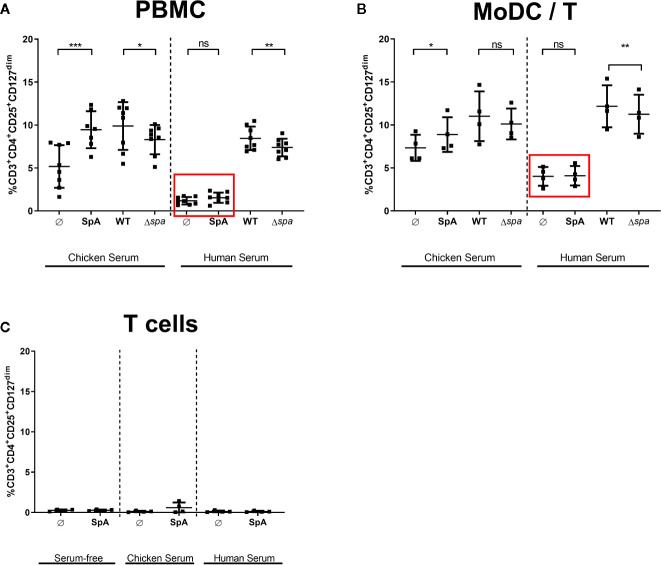
Treg induction by staphylococcal protein A (SpA) in blocked in human serum. **(A)** Peripheral blood mononuclear cells (PBMC), **(B)** monocyte-derived dendritic cell (MoDC)/T cell cocultures, or **(C)** CD4^+^ T cells only were stimulated with SpA, SA113 WT or Sa Δ*spa* for 5d in media containing chicken, human or no serum supplement (serum-free). CD3^+^CD4^+^CD25^+^CD127^dim^ cells were stained for viability and analyzed by flow cytometry. Results are shown from at least two independent experiments as single values of a minimum of four different donors with ± SD. The stars indicate statistical significance levels defined as *p < 0.05, **p < 0.01 and ***p < 0.001.

As displayed in **Figure 4**, culture in media with chicken serum resulted in higher background of Tregs in the unstimulated control. Nevertheless, SpA induced significantly more Tregs in PBMC (**Figure 4A**) as well as in MoDC/T cell cocultures (**Figure 4B**). As seen before in Ig-free serum (**Figures 2A, B**), wild type bacteria led to a higher percentage of Tregs than Sa Δ*spa*. However, this finding was only significant in PBMC cultures (**Figure 4A**). Induction of Tregs by SpA only was completely blocked compared to the unstimulated control in PBMC (**Figure 4A**) or MoDC/T cell cultures (**Figure 4B**) when human serum was supplemented in the culture media.

These results indicated that the Ig-binding sites of SpA protein might be crucial for Treg induction. Importantly, this effect was exclusively seen in the presence of APC, since no Treg induction was detected when protein was incubated with T cells only, independent on the medium (**Figure 4C**). By contrast, Treg induction by whole bacteria remained comparable in human serum, indicating that other bacterial factors contribute to activation of Tregs and that Treg induction by whole bacteria is not influenced by presence of immunoglobulins.

### *S. aureus* Induces Tregs *De Novo* in Treg-Depleted PBMC

Next, we asked whether Sa can mediate *de novo* formation of Tregs or whether it induces proliferation of the existing Treg pool. To this end, we depleted Tregs from full PBMC *via* cell sorting, stimulated with SA113 WT or Δ*spa*, respectively, and quantified CD25^+^FoxP3^+^ Tregs after 5 days (for sorting scheme, see **Figure S7**).

**Figure 5:**
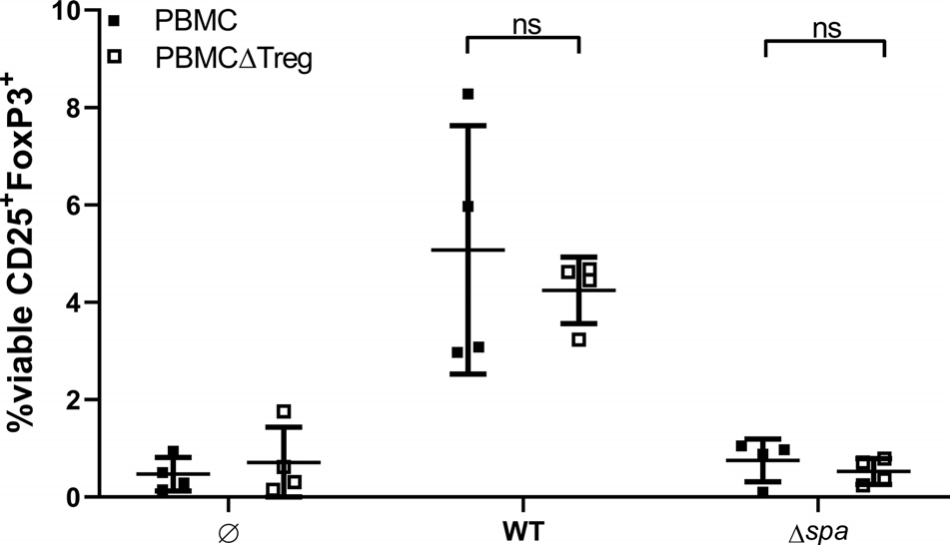
Sa restores Tregs in Treg-depleted Peripheral blood mononuclear cells (PBMC). Full PBMC (full squares) or PBMC depleted from CD3^+^CD4^+^CD25^+^CD127^dim^ cells by flow cytometry (empty squares) were stimulated with SA113 WT or Δ*spa*. After 5d, cells were re-stained and viable Tregs were detected by CD25^+^FoxP3^+^ expression. Two independent experiments with a total of n = 4 different donors are depicted as single values ± SD.

Interestingly, within 5d, the Treg population in PBMCΔTreg, left unstimulated as well as stimulated with SA113 WT was restored to approximate levels of full PBMC (**Figure 5**). However, SA113 Δ*spa* did not induce CD25^+^FoxP3^+^ Tregs in full PBMC nor in PBMCΔTreg (**Figure 5**), and neither did SpA protein (data not shown). Nevertheless, the data clearly demonstrated that SA113 WT restored Treg numbers within 5 days.

### SpA Induces MoDC-Derived Soluble Factors That Are Sufficient to Generate Tregs

**Figure 6:**
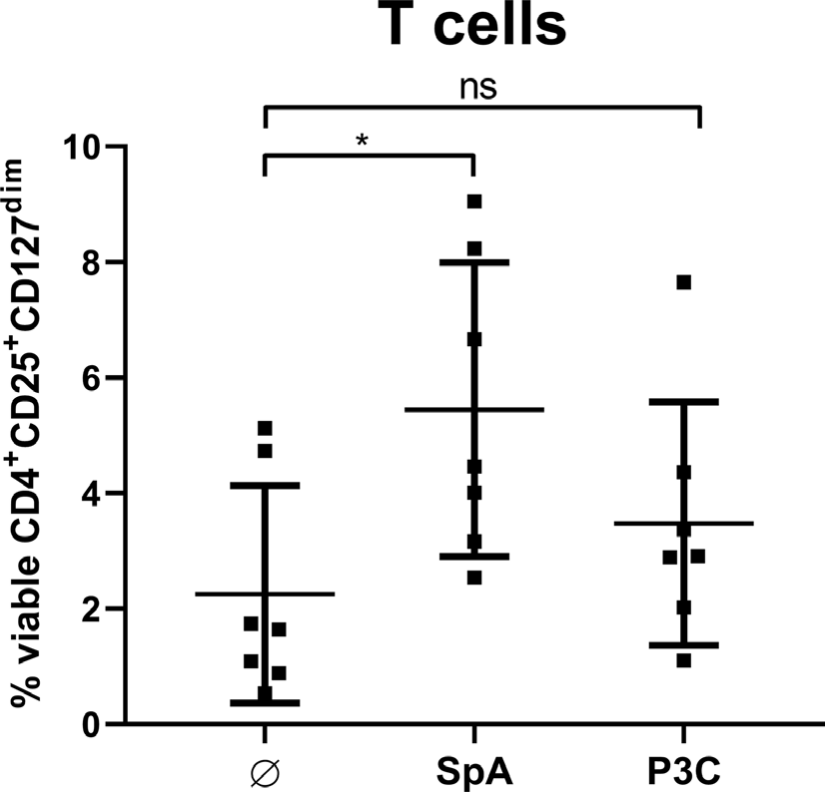
Soluble factors produced by monocyte-derived dendritic cells (MoDC) are sufficient to induce Treg. MoDC were stimulated with staphylococcal protein A (SpA) or P3C for 24 h. Their cell-free supernatant (SN) was added to autologous CD4^+^ T cells for 5d and Treg induction was measured by flow cytometry. Results are shown as values from n = 7 single donors ± SD, analyzed in three independent experiments. * indicates statistical significance level *p < 0.05.

Furthermore, we analyzed whether direct cell contact between APC and T cells is required for Treg induction. To this end, we stimulated MoDC with SpA overnight. Since *S. aureus* is known to be a strong inducer of TLR2-mediated immune responses (48), we chose the TLR2 ligand P3C, as control that leads to high levels of IL-12p40 in MoDC (49). After stimulation of MoDC, their cell-free supernatants (SN) were applied to T cells only and Treg induction was analyzed after 5 days of culture (**Figure 6**). SN of SpA-stimulated MoDC induced comparable amounts of Tregs as shown above in direct MoDC/T cell cocultures (**Figure 2B**). Induced Treg levels in the condition with SN from P3C-stimulated MoDC were comparable to the unstimulated background control. In conclusion, soluble SpA-induced factors secreted by MoDC are sufficient to induce Tregs and direct cell-cell contact between APC and T cell is not required.

### *S. aureus*-Induced Treg-Enriched Supernatant Inhibits T Cell Proliferation

Finally, we wanted to address the question whether Sa-induced Treg cultures exert suppressive activity. We stimulated MoDC/T cell cocultures with SA113 WT and collected cell-free supernatants after 5d of culture. Induction of Tregs in these cocultures with HISA was confirmed by flow cytometry (**Figure S8A**) and Treg levels upon SA-stimulation did not differ when cells were stimulated with HISA or viable SA, or in different well sizes (**Figure S8B**). Next, supernatants from Treg-enriched cultures or fresh medium were added to autologous, CFSE-labeled CD4^+^ T cells, which were subsequently stimulated with anti-human CD3/CD28 beads. Proliferation was analyzed by flow cytometry after 4 days of culture.

**Figure 7:**
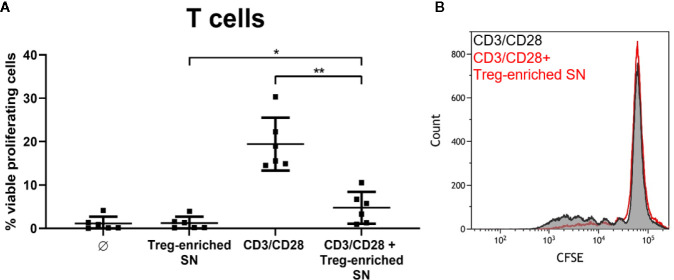
SA-induced, Treg-enriched supernatant has suppressive function. **(A)** Supernatant from monocyte-derived dendritic cells (MoDC)/T cell cocultures, stimulated with SA113 WT, was added to autologous CFSE-labeled CD4^+^ T cells. Upon stimulation with anti-CD3/CD28 beads, proliferation was analyzed by flow cytometry after 4d. Results are displayed as values from n = 6 single donors ± SD, analyzed in three independent experiments. Stars indicate statistical significance defined as *p < 0.05 and **p < 0.01. **(B)** CD4^+^ T cell proliferation upon stimulation with anti-CD3/CD28 beads ± Treg-enriched supernatant (SN) of one representative donor is shown as histogram.

Notably, addition of the Treg-enriched supernatant to CD3/CD28-stimulated T cells almost completely blocked proliferation (from 19.42% to 4.75% in average) reducing it to unstimulated level (1.2% in average). Thus, Treg-enriched SN did not induce nonspecific proliferation (**Figure 7**). Furthermore, T cell proliferation was not affected when SN from SA-stimulated MoDC cultures (without T cells) was used (data not shown). Thus, our results demonstrate suppressive activity of soluble factors released in Sa-induced Treg cocultures.

## Discussion

Due to high infestation rates and frequent exposure, the human immune system mounts an immune response but repeatedly fails to prevent Sa infection. This highlights the superb capacity of Sa to trigger immunoregulatory processes that suppress proinflammatory immune responses required for clearance of Sa (20). For instance, one of the major Sa virulence factors SpA is able to activate B cells, which leads to formation of IL-10-secreting plasmablasts and subsequent unspecific release of antibodies (31), well in line with the occurrence of nonprotective antibodies described in (50). Here, we describe a so far, unknown role of SpA in the induction of human Tregs.

Pathogen-induced control of T cell responses *via* induction of Bregs and Tregs may represent a prerequisite for colonization of the human body. A recent report highlighted a regulatory role of B cell superantigens in maintaining the balance of intestinal microbiota and immune response (51). Immune regulatory functions of SpA could, thus, support colonization of Sa in the respiratory tract.

Recent publications have highlighted other Sa virulence factors with Treg-inducing potential (41, 44–46). Here, we show that SpA is able to induce Tregs in PBMC and MoDC/T cell cocultures. Although SpA is well known as B cell superantigen, comparable Treg levels in MoDC/T cell cultures demonstrated that the presence of B cells is not required for Treg formation. Moreover, our data showed a prominent role for SpA in Treg induction because Treg induction was lower with the SA113 Δ*spa* mutant (**Figures 2A, B**). However, the effect was enhanced when whole Sa cells were used for stimulation (**Figures 2A, B**). Thus, dual stimulation *via* SpA in combination with other bacterial compounds might increase the immunostimulatory signal (52, 53). Well in line with this concept, previous studies described PSMα as an inducer of tolerogenic, Treg-inducing dendritic cells and Tregs (43, 54). Notably, costimulation with TLR4 ligand LPS enhanced this effect (41). However, experiments performed with SA113 Δ*lgt*, which lacksTLR2-activating lipoproteins, indicated that TLR2 ligands are not required for SpA-mediated Treg induction (**Figure S4**). Furthermore, absence of PSMα in SA113 strain (55) excludes synergistic activity of SpA and PSMα. However, close physical association of peptidoglycan with SpA could provide an additional stimulus supporting Treg-development (53, 56).

However, also an accessory role of other bacterial molecules such as contaminating enterotoxins cannot be excluded (44). Indeed, the SA113 strain expresses low levels of enterotoxins (57), which might be responsible for the residual Treg-inducing capacity observed with the SA113 Δ*spa* mutant (**Figures 2A, B**).

Importantly, induction of Treg was not an antigen-dependent but rather an unspecific event because SpA antigen delivered as mRNA to MoDC failed to induce Treg (**Figure 3**). Assuming that comparable epitopes were presented to T cells (20), this suggests that SpA-mediated effects occur independently of antigen-specific T cell receptor activation. This was further corroborated by the finding that cell-cell contact was not required for Treg formation (**Figure 6**): supernatant of SpA- stimulated MoDC was sufficient to induce Tregs, comparable to levels obtained in SpA-stimulated MoDC/T cell cocultures (**Figure 2B**).

Of note, residual SpA in the Treg-inducing MoDC supernatants did not directly affect Treg induction because we saw no effect of SpA on T cells only (**Figure 4C**). Also, SpA did neither induce T cell proliferation nor apoptosis in cultures of CD4^+^ T cells only (**Figure S9**). Hence, in contrast to T cell superantigens such as enterotoxins that retain activity in the absence of MHCII binding (58), SpA interaction with the APC, rather than with the T cell, was crucial for Treg induction. Nevertheless, due to its *agr*-deficiency the Sa strain used in this study does not express PSM (55) and only low levels of enterotoxins (57). This, again, argues in favor of the Treg-inducing potential of SpA.

*S. aureus* is a strong regulator of the adaptive immune response (48). Interestingly, SpA induced low levels of IL-12p70, a cytokine associated with induction of Th1 cells. However, some publications also describe a role of IL-12 in inducing Tregs and other immunosuppressive cells (59, 60). Plus, IL-12 subunits are part of the cytokine family consisting of IL-23, IL-27 and IL-35, also involved in immune regulation (61).

So far, we can only speculate whether SpA, similar to PSMα (41, 42, 54) and staphylococcal enterotoxins (62) favors the generation of tolerogenic DC (tDC) or myeloid suppressor cells, cell subsets with strong Treg-inducing capacities (63, 64). While Schreiner et al. described IL-10 and TGFβ as relevant mediators (43), Richardson et al. suggested that Indolamin-2,3-Dioxygenase (IDO), a soluble mediator with immunosuppressive function (65) as important player in Sa-triggered Treg induction. However, blocking of IDO even increased Treg numbers (41). However, as shown in **Figure S6** and published earlier (20), SpA did not induce strong secretion of proinflammatory cytokines or IL-10 in MoDC as seen with PSMα (41).

Other studies provided evidence that APC are important players in Treg induction in the context of Sa infection. Experiments carried out with cell-free supernatant of an enterotoxin-expressing Sa strain, induced expression of IL-10, IFNγ and IL-17A in FoxP3^+^ cells. This effect was decreased when monocytes were depleted from PBMC. However, in this study the main activating Sa component remained unidentified (66). When Rabe et al. blocked the interaction between PD-1 and PD-L1, the proportion of FOXP3^+^ CD25^+^ CD127^dim^ T cells was reduced upon Sa stimulation (46), highlighting the role of APC in this process. Taken together, our data complement the previous findings by providing evidence that interaction of SpA with APC is mandatory for release of Treg-inducing soluble factors. However, future work is needed to identify the driving factors.

Interestingly, the Treg-inducing potential of SpA was completely blocked in the presence of human serum (**Figure 4**). This might support the hypothesis that Ig-binding sites are crucial for Treg induction but it could also result from the presence of SpA neutralizing antibodies. Thus, we can only speculate whether the SpA Ig–binding sites interact with an APC surface receptor, or whether formation of regular or aberrant immune complexes of SpA and Ig hinders interaction with APC. Previous work suggested that the SpA Ig-binding sites are required for shedding of soluble TNF-receptor I from the surface of airway cells, leading to anti-inflammatory immune responses (67, 68). Although this was an attractive hypothesis, we were not able to confirm TNFR1 binding or engagement of an alternative receptor specific for SpA in pulldown assays and overexpression studies performed in our lab (31).

We further hypothesize that post-translational modifications of SpA such as glycosylation may also play a role in interaction with MoDC surface receptors because induction of Tregs in MoDC/T cell cocultures was not seen when cells were stimulated with recombinant SpA (**Figure S10**). By contrast, Treg induction by whole bacteria was not altered in different sera, indicating that formation of functional immune complexes does not influence Treg induction. Also, reduction of Treg levels with Sa Δ*spa* and overall Treg percentage was comparable to experiments carried out in Ig-free medium (**Figures 4A, B**). Of note, Treg induction did not depend on vitaPAMPs such as DNA or RNA, because Treg levels were comparable when cells were stimulated with HISA (**Figure S8**).

Tolerogenic DC can convert naïve T cells into suppressive Tregs (63). Depletion of CD3^+^CD4^+^CD25^+^CD127^dim^ Tregs from PBMC and subsequent stimulation with Sa WT or Δ*spa* for 5 days led to recovery of CD4^+^CD25^+^FoxP3^+^Tregs of comparable levels to baseline. We are aware that these two Treg populations are not identical and gating strategies not completely overlapping (69), which might be a reason why Treg percentages in **Figure 5** did not reach Treg levels displayed in **Figure 2A**. However, in some donors, cells were unspecifically activated through sorting, resulting in big proportion of CD25^high^ T cells. For this reason, we decided to look for expression of CD25 in combination with CD4 and FoxP3 after sort. It has been shown by others that Sa (46) and Sa superantigens (45) are able to convert CD25^-^ T cells into Tregs with regulatory function. Also Richardson and colleagues demonstrated the induction of Tregs from naïve T cells upon stimulation with PSMα (41) and Björkander et al. showed *de novo* expression of FoxP3 in CD4^+^CD25^+^-depleted T cell cultures after stimulation with cell free Sa supernatant (66).

Taken together, our data provide evidence for a novel immunosuppressory role of Sa virulence factor SpA in the induction of Tregs, including a pivotal role of APC. Importantly, our data demonstrate that Sa–induced Tregs possess strong suppressive capacities. Supernatants from Sa-derived Treg-enriched MoDC/T cell cocultures almost completely blocked T cell proliferation upon polyclonal stimulation by anti-CD3/CD28 beads (**Figure 7**).

We are aware that our data was fully acquired *in vitro*. Hence, we can only speculate whether SpA induces Tregs *in vivo* and whether this interferes with development of a protective immune response or whether it could be beneficial for the human host by blocking excessive inflammatory responses. Against this background, Sa-mediated immune suppression might represent a challenge for vaccine effectiveness. Future work should take this into account.

## Data Availability Statement

All datasets presented in this study are included in the article/**Supplementary Material**.

## Author Contributions

JU and IB-D designed the study and planned the experiments. JU, KH, and OT performed and analyzed the experiments. JU, OT, and IB-D interpreted the results. JU and OT prepared the figures. JU and IB-D wrote the manuscript.

## Funding

This research was funded by the Paul-Ehrlich-Institut. It received no specific grant from any funding agency in the public, commercial, or not-for-profit sectors.

## Conflict of Interest

The authors declare that the research was conducted in the absence of any commercial or financial relationships that could be construed as a potential conflict of interest. 
