# Supplementary material for: Staphylococcus aureus Protein A Induces Human Regulatory T Cells Through Interaction With Antigen-Presenting Cells
Source: Front Immunol. 2020 Sep 30;11:581713. doi: 10.3389/fimmu.2020.581713 (PMC7560526; doi:10.3389/fimmu.2020.581713)
Supplement: Supplementary file 1 [file DataSheet_1.pdf]

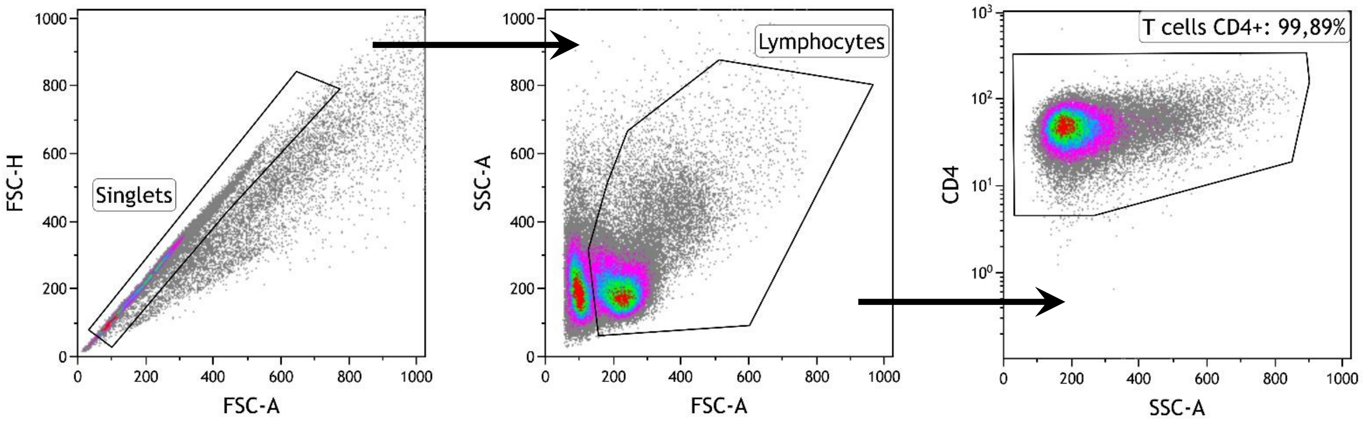

Fig. S1 Purity of CD4⁺ T cells isolated via AutoMACS. Cells were gated for singlets, viable lymphocytes and CD4⁺ cells. Dot plots of one representative donor are shown.

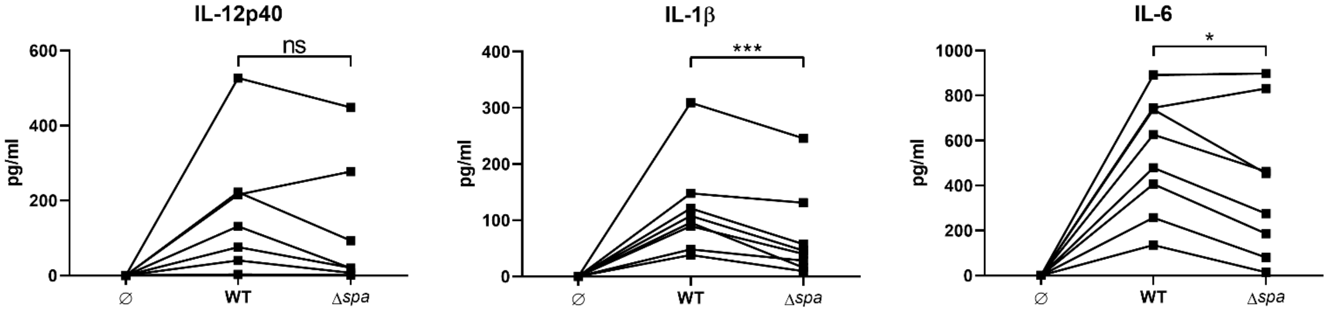

Fig.S2 SpA induces T cell cytokine secretion. MoDC / CD4$^+$ T cell co-culture were stimulated with SA113 WT or the *spa*-deficient mutant (Δ*spa*). Cytokines levels were analyzed supernatants harvested on day 5. Single values (dots) from n=8 different donors form four independent experiments are displayed.

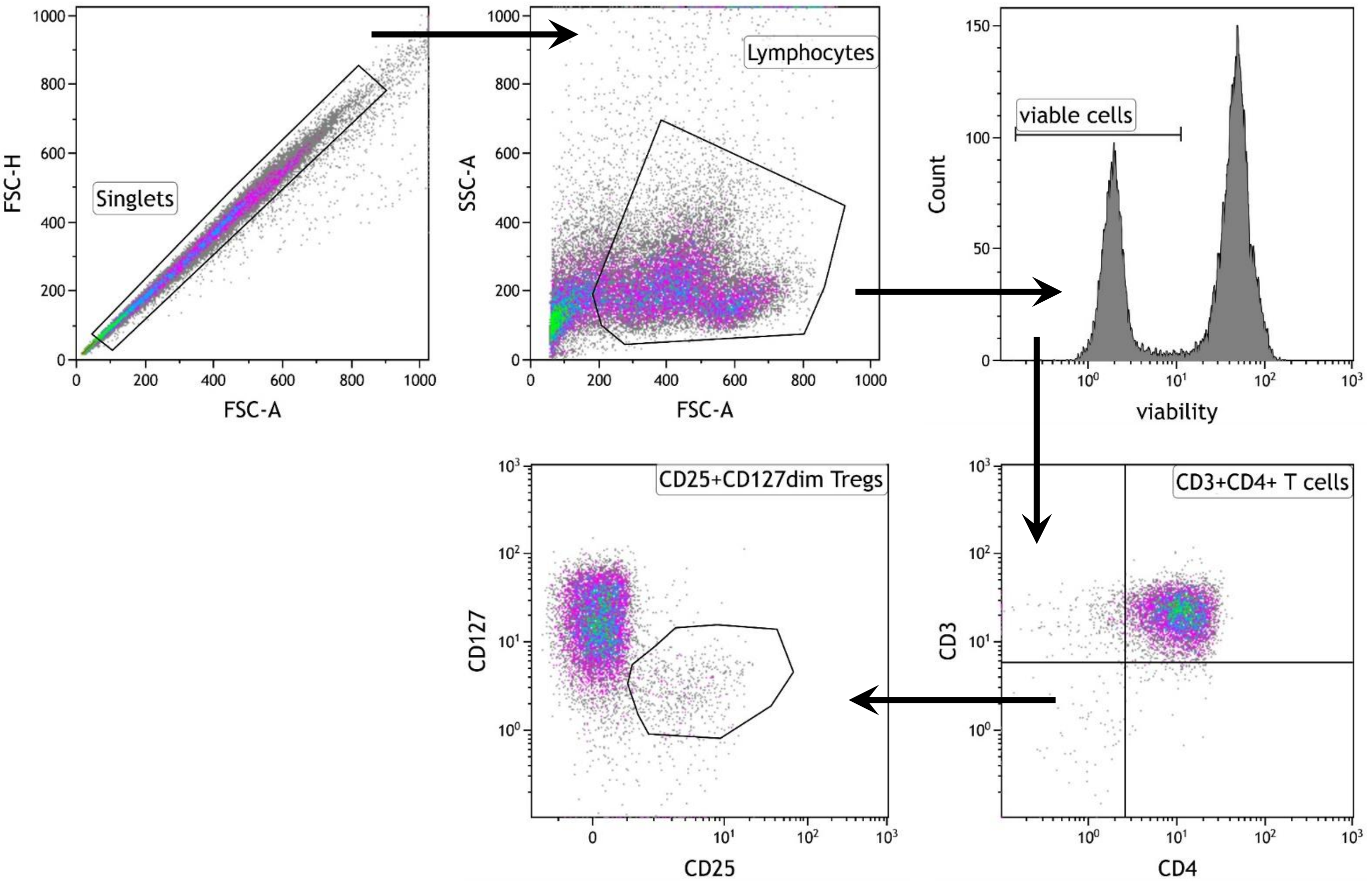

Fig. S3 Treg gating strategy. Cells were gated for singlets, viable lymphocytes and $CD3^+CD4^+$ cells, followed by $CD25^+CD127^{dim}$ expression. Dot plots and histograms of one representative donor are shown.

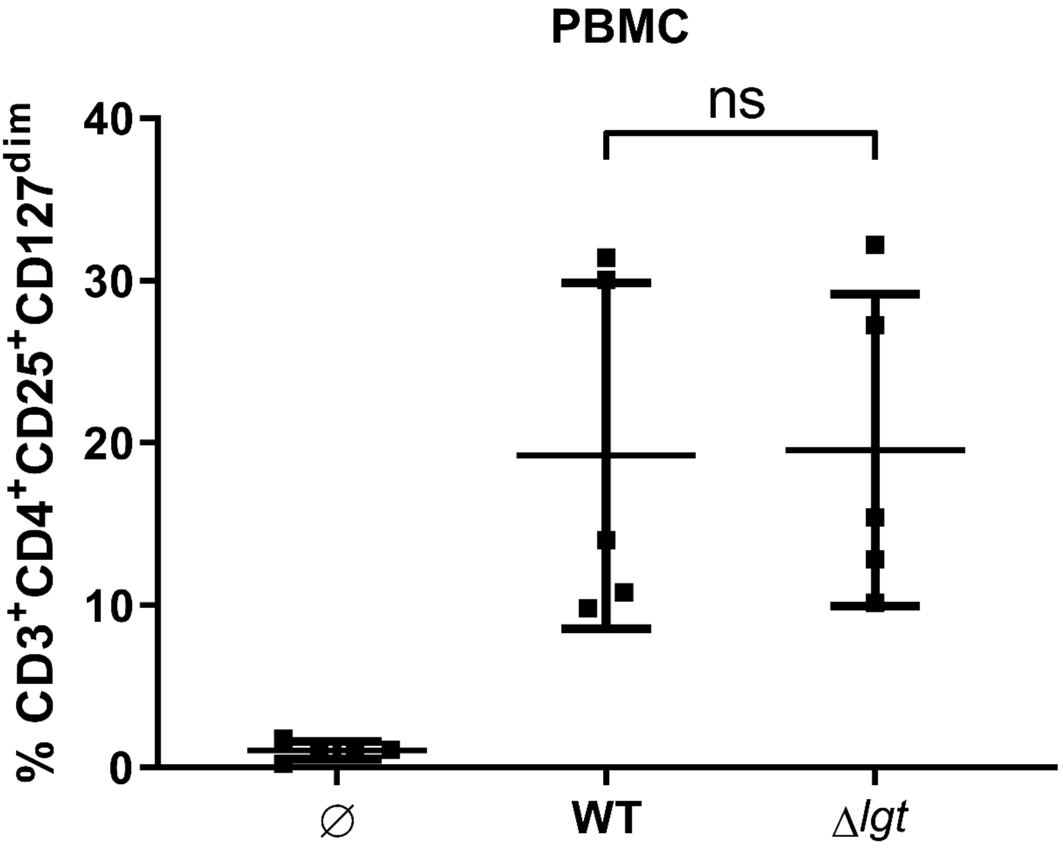

Fig. S4. Absence of TLR2-active lipoproteins has no effect on Treg induction. PBMC were stimulated with SA113 WT or Δ*lgt*, lacking TLR2-activating lipoproteins. After 5d cells were stained for viability and $CD3^{+}CD4^{+}CD25^{+}CD127^{dim}$ and analyzed by flow cytometry. Results are shown from two independent experiments as single values of n=5 different donors with ± SD.

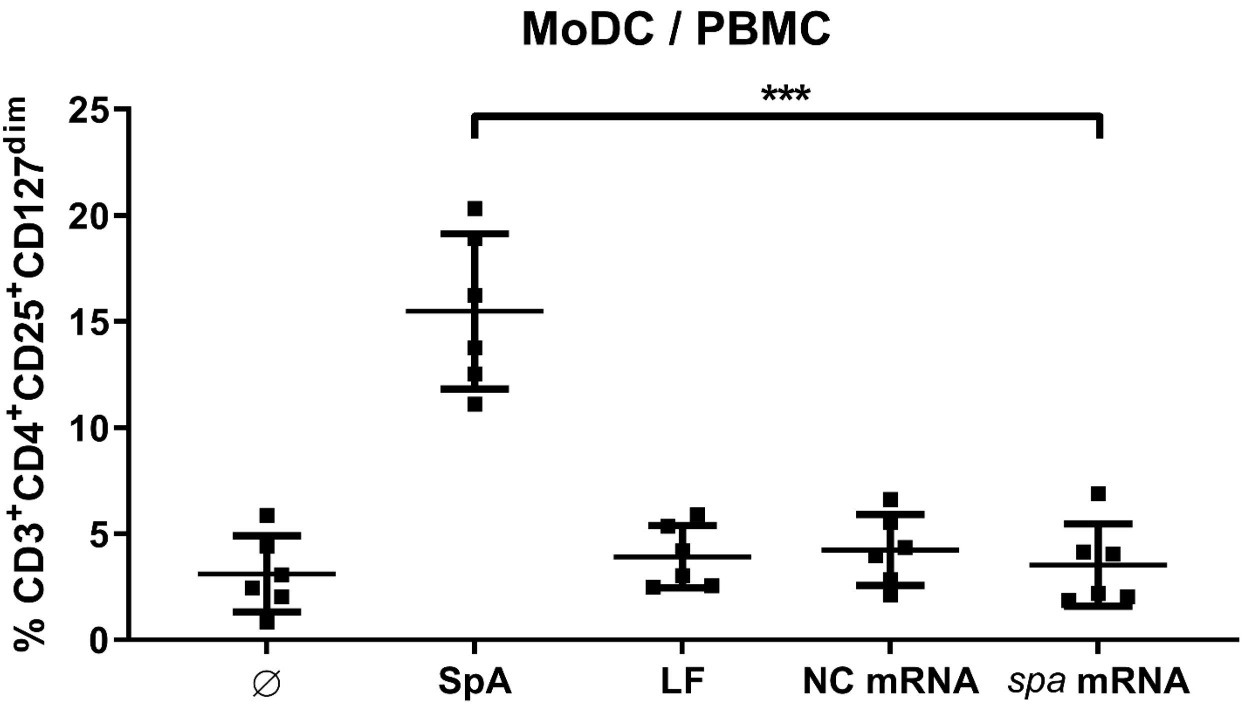

Fig. S5 Native protein conformation of SpA is mandatory for Treg induction. MoDC were stimulated with SpA or transfected with *ivt* mRNA encoding *spa* or with non-coding control mRNA (NC) and added to PBMC. Lipofectamine (LF) was used as transfection control. After 5d, cells were stained for viability, $CD3^{+}CD4^{+}CD25^{+}CD127^{dim}$ and analyzed by flow cytometry. Results are shown from three independent experiments as single values of n=6 different donors with ± SD.

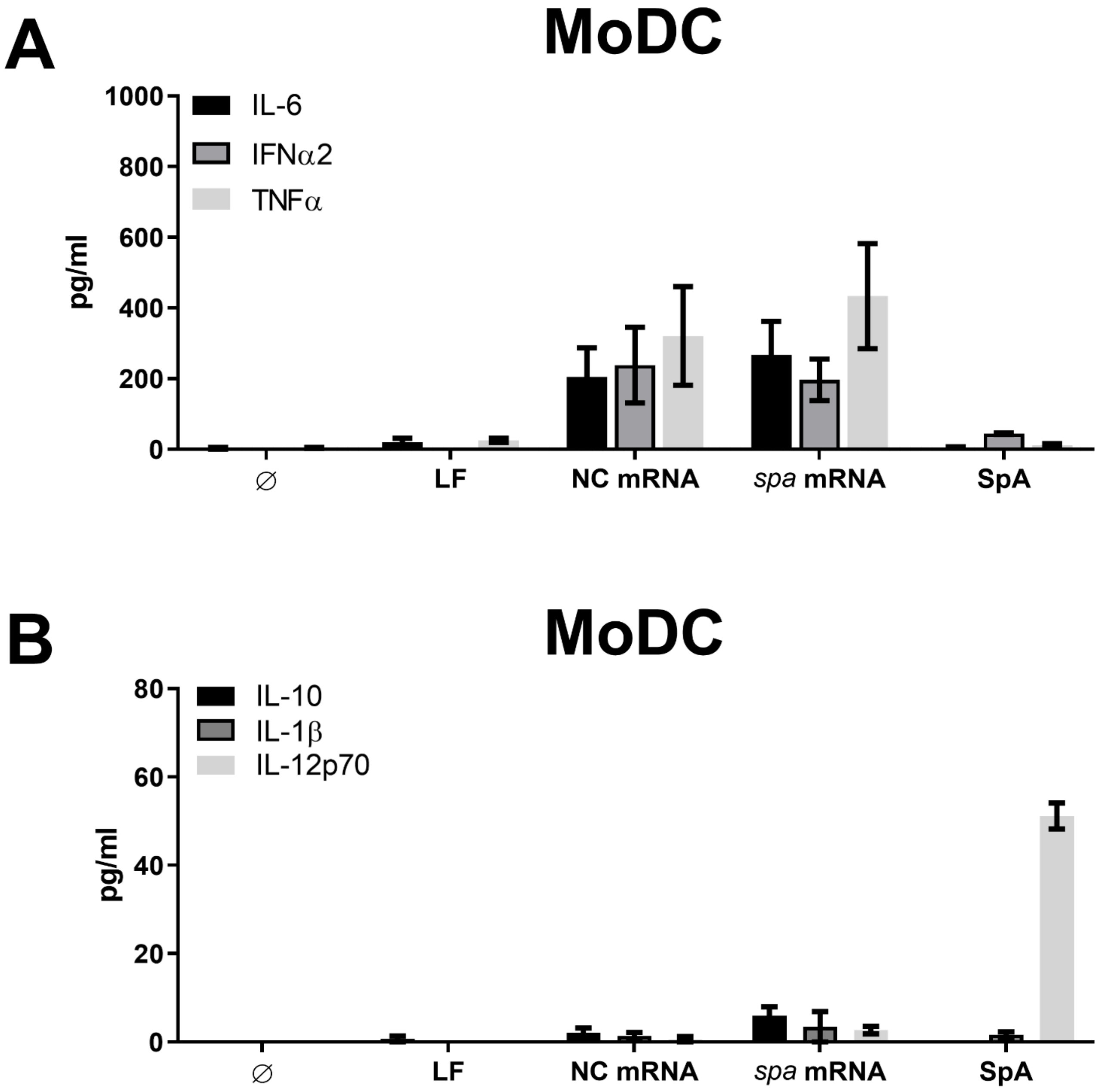

Fig. S6 mRNA induces cytokine secretion in MoDC. MoDC were stimulated with SpA protein or transfected with *spa* encoding or control non-coding mRNA (NC mRNA). LF served as transfection control. Induction of **(A)** IL-6, IFNα2, TNFα and **(B)** IL-10, IL-1β and IL-12p70 was assayed after 24 hours by Multiplex Cytokine Assay. Results are shown from three independent experiments of n=6 different donors with ± SD.

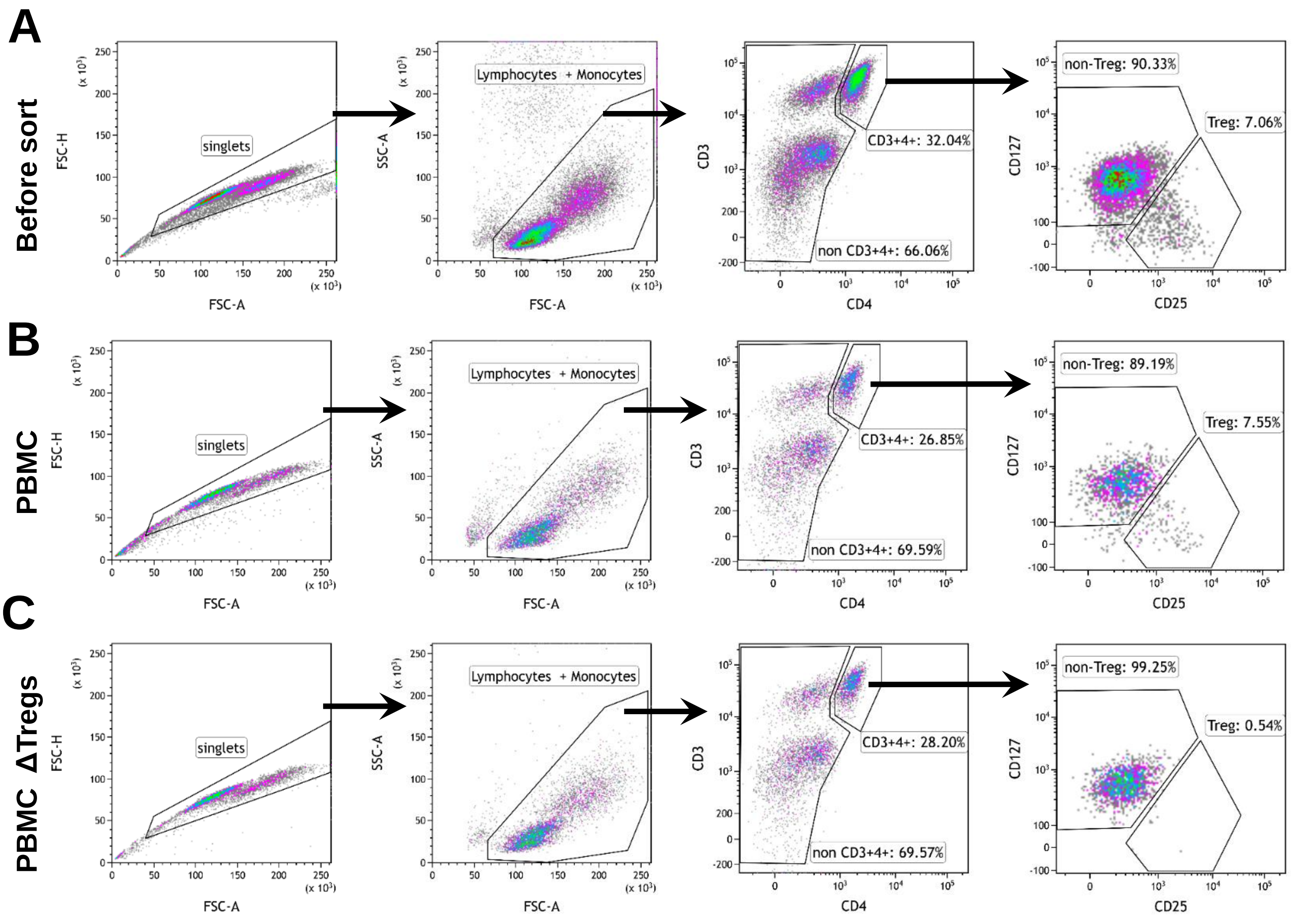

Fig. S7 Depletion of Tregs. (A) Gating strategy for cell sorting**.** Cells were gated for singlets, lymphocytes and monocytes, $CD3^+CD4^+$ cells, followed by $CD25^+CD127^{dim}$ expression for Treg detection. PBMC depleted from Tregs from same donors were sorted as non $CD3^+CD4^+$ cells together with non-Tregs. In subsequent sort the full PBMC were sorted from gate „Lymphocytes + Monocytes“ as a control. **(B)** Re-analysis of full PBMC and **(C)** Re-analysis of PBMC depleted from Tregs. Dot plots of one representative donor are shown.

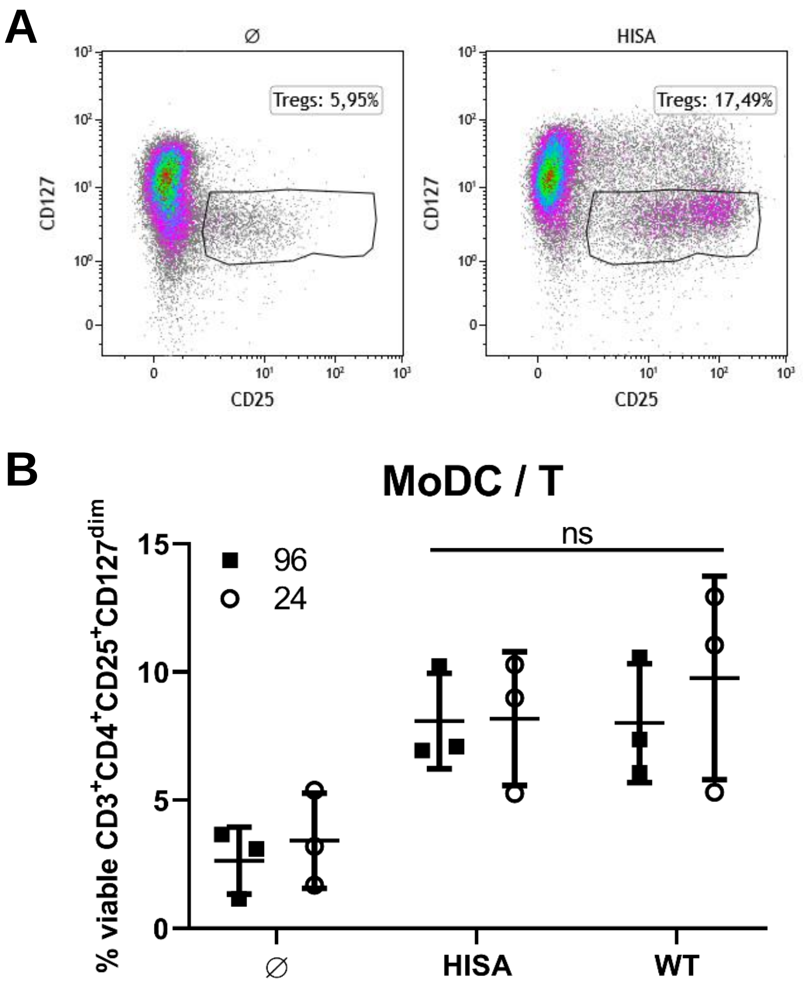

Fig. S8 Treg induction by heat-inactivated SA113 WT (HISA) is comparable with viable SA. (A) Viable $CD3^+CD4^+CD25^+CD127^{dim}$ Treg induction upon stimulation of MoDC / T cell co-cultures with HISA (MOI 10) in 24 well plates for 5 d was confirmed by flow cytometry. Dot plots of the unstimulated control (Ø) and after stimulation with HISA are shown of one representative donor. **(B)** Comparison of Treg levels in MoDC / T cell co-cultures upon stimulation with HISA or viable SA113 (WT) in 96 well plates or 24 well plates after 5d shows no differences. Experiments were carried out in medium supplemented with human serum for 5d. Results are shown as mean ± SD of single values of n=3 donors, analyzed in two independent experiments.

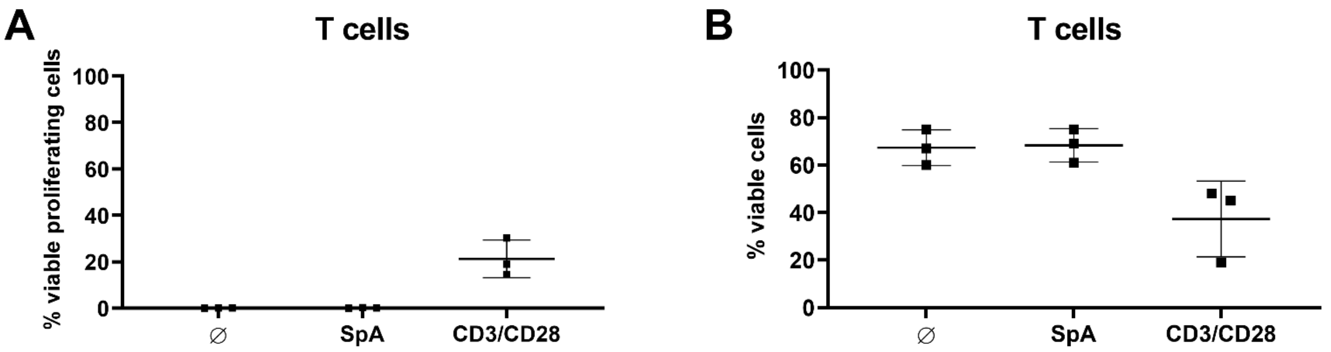

Fig. S9 SpA does not induces T cell proliferation or T cell death. (A) $CD4^+$ T cells only were stimulated with SpA or anti-CD3/CD28 beads as postitive control for 4d to address proliferation. Proliferating, $CFSE^{low}$ cells **(A)** were detected after CFSE staining, **(B)** viable cells after Live/Dead staining by flow cytometry.

Results are shown from n=3 different donors from one experiment as single values and the mean ± SD.

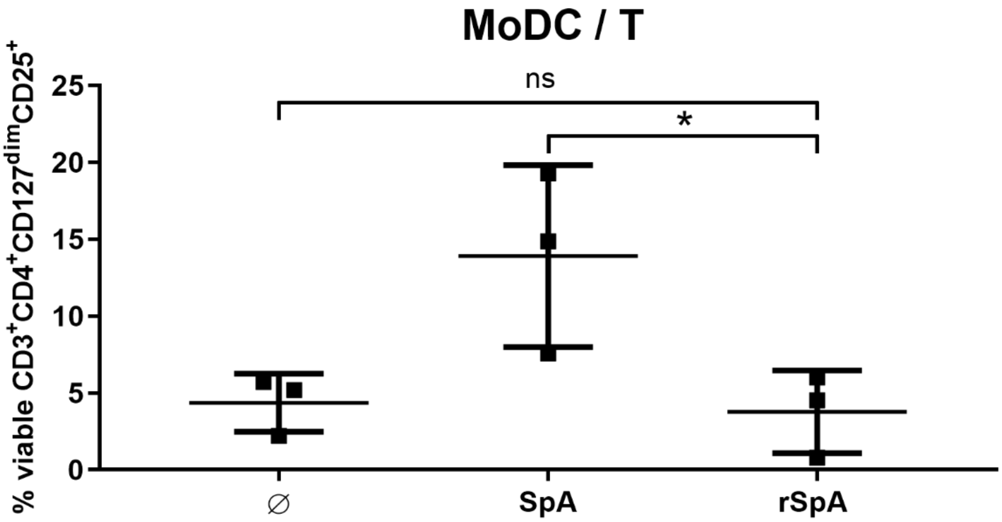

Fig. S10 Recombinant SpA does not induce Tregs. MoDC / T cell co-cultures were left unstimulated (Ø) or were stimulated with 1 µg/ml SpA from SA or recombinant SpA (rSpA), respectively. Treg levels were determined by flow cytometry after 5d of culture. Treg induction by rSpA remained at unstimulated levels. Results were displayed as single values and the mean ± SD of n=3 different donors, performed in two independent experiments.
